# Philadelphia Academy of Surgery

**Published:** 1886-03

**Authors:** 


### SOCIETY REPORTS.

PHILADELPHIA ACADEMY OF SURGERY.

*At a stated meeting held November 2, 1885*, D. HAYES AGNEW, M. D., President of the Academy, in the chair, a communication entitled

NOTES ON THE NEW ANTISEPTICS, HYDRONAPHTHOL AND THE POTASSIO-MERCURIC IODIDE, WAS READ BY R. J. LEVIS, M. D., one of the Attending Surgeons to the Pennsylvania Hospital.

The following are the claims made for the newly-discovered antiseptic, hydronaphthol:

It is at least twelve times as effective as carbolic acid, and is entitled as a true antiseptic to occupy a position in the comparative tables next to the mercuric bichloride.

It is thirty times as potent as salicylic acid, sixty times as effectual as boric acid, and has six hundred times the antiseptic power of alcohol.

Hydronaphthol is soluble when placed in cold water to the extent of one part in two thousand. It is soluble in hot water in the proportion of one to one hundred; but when the water becomes cooled to ordinary temperatures a precipitate occurs, leaving a solution of one to one thousand. In this strength of one to one thousand it permanently prevents the development of the germs of putrefaction in all putrescible fluids.

Whilst the true antiseptic or *inhibitory* action of hydronaphthol in such cold aqueous saturated solution is perfect, its germicidal and proper disinfectant power is ineffective. For the destruction of already existing germs, such as have a tenacious vitality, as those of anthrax bacilli and pathogenic micrococci, it therefore cannot be relied on. As to its action in this regard, as compared with carbolic acid, it should be remembered that a ten-per-centum carbolic solution is required,—a strength practically improper in wound-treatment. In ordinary antiseptic practice, carbolic acid is valuable only on account of its inhibitory action.

The first use of hydronaphthol as an antiseptic was by Dr. G. R. Fowler, of Brooklyn, to whom the profession is indebted for its introduction to practical surgery. My own experience with the antiseptic action of hydronaphthol in the wards of the Pennsylvania Hospital and in private surgical practice confirms his observations, as given in his recent articles in the *New York Medical Journal*.

Hydronaphthol is a grayish substance, in the form of crystalline lamina, having a slightly aromatic taste and odor. It is soluble freely in alcohol, ether, chloroform, glycerin, benzole, and the fixed oils.

In the aqueous saturated solution of one to a thousand it is absolutely unirritating, and has no toxic action, either local or systemic; is free from unpleasant odor, and has no injurious action on metallic instruments or on clothing fabrics.

Besides its use in aqueous solution, I have used it in the form of a powder diluted, preferably with the oxide of zinc in the proportion of one to fifty.

I believe that hydronaphthol may well displace carbolic acid from practical surgery.

The potassio-mercuric iodide is four or five times as powerful as a true germicide or disinfectant as the mercuric bichloride. For such use it is effective in aqueous solutions in the proportions of only one to twelve thousand.

The potassio-mercuric iodide is made by simply dissolving equal quantities of the biniodide of mercury and the iodide of potassium in distilled water. The solution is evaporated, and there remain yellow, needle-like crystals of the potassio-mercuric iodide.

In the use of such dilutions of this powerful antiseptic, local irritation is entirely avoided, and the risk of producing the constitutional effects of mercury is greatly diminished.

The introduction into surgical treatment of these two remarkable and powerful substances, hydronaphthol and the potassio-mercuric iodide, will do much to overcome some of the objections and inconveniences of antiseptic practice.

DISCUSSION.

Dr. S. W. Gross: Dr. Levis has made the statement that the potassio-mercuric chloride is a far safer germicide than the ordinary mercuric chloride. The solution of one to twelve thousand is really not so much weaker, if we look at it properly. The potassio-mercuric chloride is dependent for its activity on the biniodide of mercury. It is a well-established fact that the biniodide of mercury is a far more powerful germicide than is the bichloride. Because we use a weaker solution is no evidence that it is not as strong as the bichloride. I can see no advantage in making the potassio-mercuric chloride solution, unless it is to fix the biniodide. In preparing the solution of corrosive sublimate in which we wish to keep gauze, sponges, etc., for a long time, we add to it seven and one-half grains of chemically pure chloride of sodium, with the view of fixing the bichloride so that it will not be converted into calomel. The addition of an equal part of iodide of potassium to the biniodide will fix that salt so that it will not be decomposed. There is therefore really no advantage in it, except to prevent changes in the biniodide.

Hence this is not a new remedy, for the biniodide has been used as a germicide. I do not see the advantage of using a stronger substance in what is apparently a weaker solution. It is of course impossible to say anything as regards the constitutional effects, for Dr. Levis has not had sufficient experience to determine whether or not toxic symptoms are produced by this agent. There is no reason why,—if the potassio-mercuric chloride is used as carelessly as the corrosive sublimate often is,—there should not be toxic symptoms produced. If the cases of poisoning with the bichloride of mercury which have been reported are examined, it will be found that the bichloride has been used in unusually large quantities. For example, a one to one thousand or one to two thousand solution has constantly been used as a fluid to irrigate the wound during a surgical operation. Again, in psoas and iliac abscess, where a large quantity of the solution has been introduced after the evacuation of the pus, toxic symptoms have arisen. If a little care is exercised, there is no reason why toxic symptoms should arise from any of the mercurial solutions.

In regard to hydronaphthol, I know nothing of it from experience. I, however, know that Dr. Fowler has been making experiments with it for years, and, even after the adoption of mercurial solutions, used saturated compress with naphthaline outside of the corrosive-sublimate dressings to keep the wound enveloped in the vapor of naphthol, according to his statement.

Dr. R. J. Levis: Dr. Gross will bear in mind that with the potassio-mercuric chloride there is only one-fifth the amount of mercury used. We know that the mercuric bichloride is an unstable salt in the way in which it is generally used, and for ordinary uses it can hardly be made a stable salt.

Dr. S. W. Gross: It is a stable salt. A solution of corrosive sublimate in water can be kept for a week without any change. To make a stable solution for sponges and dressings, it is better to add chloride of sodium.

Dr. R. J. Levis: I used the term unstable with reference to the mercuric chloride in contact with organic matters. Under such circumstances it is liable to be converted into ordinary calomel.

Dr. S. W. Gross: This is a mistake. Corrosive sublimate does not become unstable in the presence of organic compounds. This has been asserted, but there is no proof of it. If it did undergo this change, why do we have toxic symptoms arising when large quantities of the solution are injected into abscess-cavities?

Dr. J. M. Barton: As regards stability, the mercuric chloride does not seem to undergo any chemical change. At the same time, it undergoes some change when brought in contact with organic matter. If this were not so a large mass of odorous material could be disinfected with half a grain of corrosive sublimate.

Dr. Charles W. Dulles: It is well known that the salts of mercury in the presence of albumen are apt to be converted into albuminoids, but this does not prevent the constitutional effect of mercury. I believe that this change is not in sufficient quantity to interfere with the antiseptic properties of the substance. The effect of the potassic mercuric chloride in the presence of albumen is somewhat similar. Dr. Oliver, who has made some extensive experiments in regard to albumen in the urine, has found that this potassio-mercuric chloride is the most delicate test for albumen.

Dr. S. W. Gross: In corroboration of what Dr. Dulles has said, I would recall the fact that when Lister began the use of corrosive sublimate he employed a solution which was too strong, and found that a certain amount of erythema and vesication was produced. He now procures the serum of the blood of a horse and makes his solution in that way.

Dr. Addinell Hewson: I have had some experience with hydronaphtol, but I have seen it produce irritation, and even such erythema as was alarming. This was a solution of one to two thousand. The effects were produced within twenty-four hours. The patient experienced constant distress from the time of its application.

Dr. R. J. Levis: I have found the one to two thousand solution almost tasteless. I have placed it in one eye without being conscious of its presence.

Dr. Addinell Hewson: In this case there was a tendency to erythema, and the result undoubtedly proved that the remedy had no effect as a germicide.

Dr. Levis: That is not claimed for it.

Dr. F. H. Gross read a paper on *A Suture for the Approximation of Deep Wounds.*

Dr. R. J. Levis: I wish to call attention to a suture to be used in place of the buried suture. Every surgeon recognizes the advantages of approximating the surfaces of wounds as conducing to healing. We have all been too much satisfied with approximating simply the edges of wounds without paying any attention to the approximation of the deeper parts. In amputations and in the removal of tumors from such situations as the groin and axilla, large spaces are left which may become filled with blood, serum, or the products of inflammation. Since the introduction of antiseptic surgery, and particularly since the use of animal sutures has been adopted, some surgeons have used what are called buried sutures. These have their disadvantages, and sometimes it is quite difficult to apply these sutures so as to secure complete approximation.

Some time ago I devised a simple plan of approximating the deeper parts of a wound. Taking an amputation-wound for illustration, to approximate the flaps in this manner I take an ordinary silver wire armed at each end with a straight needle, introduce one of the needles into one flap at the bottom of the wound and bring it out about half-way between the angle of the wound and the edge of the flap.

It is then carried across the wound and through the opposite flap. The second needle is then carried through in the same manner. A figure of 8 is thus formed. When the ends of the wire are drawn upon, the deeper portion of the wound is approximated. The superficial portions of the wire are then carried across the surface and bring together the edges. Superficial sutures are also introduced. I have used this in a number of amputations, and think it particularly applicable to the closure of wounds after the removal of large tumors of the breasts where the axillary glands are also removed. In a case in which I removed the breasts four days ago there is now complete union. I insert as many of these sutures as may be necessary. I have always used wire, but ligature rendered antiseptic would probably be almost as good.

Dr. J. Ewing Mears: In deep wounds of the axilla, what tissues do you approximate?

Dr. Levis: Fatty tissue, muscular tissue, or whatever may be necessary.

Dr. Addinell Hewson: I have used the suture of Dr. Levis frequently, but I have used it without the addition of superficial sutures. I employ, instead, collodion and gauze. This I find more satisfactory. I have had direct union through a stump without suppuration whatever on more occasions than one.

Dr. S. W. Gross: I have seen Dr. Levis apply this suture, and it does approximate wounds in a most excellent manner. It is very easy to get the suture out. It is very desirable to get rid of the presence of any foreign body in the wound. Neuber, the introducer of the decalcified-bone tubes, recognized their inconvenience, as well as the disadvantages of the ordinary rubber drainage-tube. It was he who proposed buried sutures. He also resorted to other means of drainage. In amputation of the breast he occasionally punched holes in the skin to allow free drainage. In removal of axillary tumors he inverted the skin on each side, attaching the anterior portion to the pectoralis major and the posterior to the latissimus dorsi, thus making a funnel-shaped opening through which drainage could be effected.

Dr. T. G. Morton: During last winter, something similar to this was proposed by Dr. Packard at the Pennsylvania Hospital. He suggested the introduction of a long pin across the stump, and then the application of a figure of 8 around, making careful compression. This would answer very much the same purpose.

Dr. R. J. Levis: This suture might be called the duplex suture or the deep-crossed suture. I think it would be very suitable in the high operation for stone, the deep portion of the suture including the bladder and the superficial portion including the superficial tissues.

PIN FOR TREATMENT OF FRACTURE OF THE PATELLA.

Dr. Morton: I wish to exhibit a pin which I have used in the treatment of fractured patella. For a number of years I have been using the ordinary Malgaine's hooks and the modified Malgaine's hooks. It occurred to me that if I could pass a steel pin and keep the fragments approximated I should have an easier method than with the hooks. I devised this pin, which has a detachable handle and works as an ordinary Brainard's drill. In the case in which I applied it the pin went through without difficulty. Over the extremities shoulders were applied. The fragments were then not disturbed for three weeks. There was not the slightest irritation. Lead-water and laudanum were kept applied. When the pin was removed, I found under one of the shoulders a small collection of pus. In order to obviate this I have had a shoulder made which does not come in contact with the skin at a right angle. To avoid the possibility of pulling any pus into the bone when removing the pin I have made it in two pieces, united in the middle with a screw. When it is desired to remove the pin, the parts are unscrewed and the two pieces removed. I have had less trouble with this than with any other method of treating fractured patella.

In introducing the pin I did not employ any anæsthetic, and the patient made no complaint. The whole manipulation did not occupy over fifteen seconds. It is impossible to apply Malgaine's hooks without an anæsthetic. I was surprised at the ease with which the pin passed through the bone. It met with no more resistance that it would in going through a hard Boston cracker.

I was unable to apply the pin until thirteen days after the fracture, on account of the effusion within the joint. I have never put on the hooks until all local irritation has subsided.

In this case I do not think that there is bony union. I have never seen bony union where there has necessarily been delay, but there is here exceedingly close union.

Dr. Wm. W. Keen reported a case of *Obstructive Jaundice, Cholecystotomy for Impacted Gall-Stones.*

Albert Henry K., German, aged 45, residing in Philadelphia, was admitted into the hospital September 21, 1885, during the service of Dr. W. W. Keen. Parents healthy.

He is the only living one of twelve children; the rest died during infancy.

General health was good, with occasional attacks of gastro-intestinal catarrh. Four years ago he had a severe attack of colic, the pain arising in the right hypochondriac region and extending to the umbilicus. The attack lasted several hours, and was relieved by medicine. The following year he had a similar seizure, followed by a chill, loss of appetite, vomiting, and constipation, and in a short time the conjunctiva was jaundiced.

He had another attack June 12, 1884, followed by nausea, vomiting, chill, loss of appetite, constipation, high-colored urine and clay-colored stools, with marked jaundice of his whole body, which continued ever since. Since that time the attacks have become more frequent, occurring once or even twice weekly.

He had been, since January 1, 1885, under medical care, but with no improvement. He first came to the dispensary August 6, and was under observation and treatment, but without any change for the better.

Upon admission, it was noted that he had lost some flesh; his mind was dull; the skin, conjunctiva, and mucous membrane were intensely jaundiced; marks of scratching appeared on the abdomen, and he suffered much from itching and stinging at night; the pulse was 54 and full; tongue slightly coated with yellow fur in the morning. His appetite was poor, bowels costive, and the stools were of a clay color. He also complained of flatulence and constant tenderness and distress in the epigastrium. The urine was high-colored, acid, sp. gr. 1025; no albumen was detected, and no sugar; bile-pigment was present.

Physical examination showed a tumor below the margin of the ribs in the right hypochondriac region; it extended from the right nipple-line three and a half inches to the median line, and to within two and a quarter inches of the umbilicus. It was not absolutely flat upon percussion, but tender and elastic. The liver-dullness extended from the sixth interspace in the nipple-line to one-fourth inch below the margin of ribs. The abdomen was not distended; spleen of normal size; lungs and heart both normal.

September 24. Had severe headache; pain in the epigastrium, followed by a rigor lasting three hours, then fever. 5 P.M. Temp. 101.5°; pulse 80; podophyllin (gr. ½ at night) acted freely on the bowels.

September 28. Dr. Keen introduced into the tumor a hypodermic needle, and thought he detected a stone. A very small quantity of liquid was withdrawn; it was dark-colored and mostly blood.

October 1. An aspiratory needle was introduced into the tumor three inches, and a probe passed through it. A stone was detected.

October 4. He had a chill, which lasted three hours, followed by the usual symptoms. The operation which was appointed for the next day was, therefore, postponed.

October 10. Dr. Keen, assisted by Drs. Grove, Mears, O'Hara, Willetts, Elmer, Learned, Boyd and others, operated, with the usual antiseptic precautions, including the spray, carbolic acid being used.

The surface of the body was first washed off with carbolized water; etherization was commenced at 1.30 P.M. An incision of three inches was made parallel with the margin of the ribs, commencing one and a half inches below the ensiform cartilage, and extending over the prominence of the tumor. All the vessels were tied with catgut ligature. The abdominal walls were thick. All hæmorrhage had ceased before the peritoneum was opened. Now the liver came into view, and what was thought to be the tumor—the distended gall-bladder—proved to be an enlarged left lobe of the liver, presenting much more to the right than usual. The gall-bladder could neither be seen nor felt.

In order to obtain more room, the incision was prolonged about two inches to the right. The colon and omentum were now exposed. Still the gall-bladder could not be detected with certainty; but a hard mass could be obscurely felt, which was believed to be gall-stones. It lay transversely, directly in front and slightly to the right of the spine, and almost absolutely in contact with it. To obtain still more room to work at such a depth, another incision was made, beginning at the upper end of the first incision, and extending downwards in the linea alba three inches. The closest search was made for the gall-bladder, being still in doubt as to its situation.

From the posterior part of the transverse fissure, almost in contact with the spine, an apparently uniform mass, continuous with the gastro-hepatic omentum, extended downwards and forwards, in the posterior part of which, as stated, the gall-stones could be somewhat obscurely felt near the liver; and, farther away from it, a softer mass, which ought to be the flaccid gall-bladder. These lay in such relation to each other that the gall-stones were thought to be in the cystic and the common duct.

Into this softer mass—the supposed gall-bladder—a small opening was made; but it was soon evident that this was the duodenum, as the finger detected the pylorus. Discovering this, the wound in the duodenum was closed with five interrupted and five carbolized-silk Lembert's sutures. The harder mass containing the gall-stones was now carefully lifted to a slight extent by the finger and thumb, and the wall and tissues in front of it cautiously incised for about three-fourths of an inch, and two stones, respectively three-fourths and one and one-fourth inches in size, removed. The gall-bladder was as firmly contracted around them as a kid-glove clasps the finger, and contained absolutely no fluid. The opening of the cystic duct could be felt by the finger, and was patulous. No other stones could be felt. A probe, it was thought, could be passed from the cyst to the common duct.

The wound in the gall-bladder was stitched with four interrupted and four Lembert's carbolized-silk sutures. All hæmorrhage had apparently ceased. The cavity was carefully cleansed, and the abdominal walls, including the peritoneum, were sutured with wire, and the wound dressed with bichloride-of-mercury gauze. The operation lasted until 4.45 P. M. His pulse during the operation was never more than 75, nor under 60, and his skin was fairly warm; but his respiration, from early in the operation, was sighing.

After the operation, he suffered from moderate shock. Hot bottles were applied along his extremities.

5 P. M. Temperature, 99.5°; pulse, 88. He suffered very much pain and complained of thirst. Morphine, gr. $\frac{1}{8}$, and cracked ice.

5.30 P. M. Hiccough; relieved by atropia, gr. $\frac{1}{120}$.

7 P. M. Pulse 96, and compressible; temp., $99\frac{4}{5}$°. Morphine, gr. $\frac{1}{6}$. Small quantities of brandy and water were given at intervals.

9 P. M. Feeling quieter. Still complains of pain.

11 P. M. Pulse, 104; respiration, 24; morphine, gr. $\frac{1}{8}$, was given at intervals; through the night he gradually failed.

October 11. At 7 A. M., pulse very feeble and rapid; temp.

$101\frac{2}{5}°$. Digitalis and brandy were given; but he did not rally, and died at 8 A. M.

The post-mortem examination was made at 12 M. Rigor mortis well marked, though the trunk was still warm. All the abdominal contents were deeply jaundiced. About six to eight ounces of blood were found in the belly, and, as the greater part of it was behind the peritoneum and extended to and around the right kidney, it presumably came from vessels injured in the lifting of the mass in which the gall-stones were situated. This had been carefully done by Dr. Grove, as it was impossible to obtain access to it otherwise at such depth, but required some force. No hæmorrhage had been observed at or immediately after the operation. There had been no bleeding from the operation-wounds, either in the shrunken gall-bladder, the walls of which were very thin, or from those in the duodenum or the abdominal walls. There was no hæmorrhagic tendency noticed, as is so often the case in such operations. There also had been no escape of bile or intestinal contents into the peritoneal cavity. No organs were observed except those concerned in the operation.

The liver, gall-bladder, stomach, duodenum, pancreas, and colon were removed in mass, and showed the following facts:

The left lobe of the liver, especially its anterior portion, was much enlarged and somewhat twisted to the right, so that its semi-lunar projection occupied the position of the gall-bladder, and was easily mistaken for it before the operation. The liver was deeply fissured between the right as well as the left lobe and the lobus quadratus. No gall-bladder existed in the proper site for it, but it was found lying transversely in a shrunken condition, as stated, far posteriorly. The common and cystic ducts were patulous. The wounds in the gall-bladder and the duodenum were effectively closed by the sutures, and no escape of the contents had taken place. No other gall-stones were found. The mucous membrane of the duodenum was deeply congested for a wide extent, undoubtedly from inflammation existing before the operation. The duct of the pancreas was pervious.

So far as I am aware, such complications as this displacement of the gall-bladder, its shrunken condition, and the impossibility of deciding what was and what was not gall-bladder have not before been reported. It may be said that the incision should have been first made at the place where the stones were felt; but it was decided not to do so on account of the obscurity with which they could be felt lying under considerable tissue, which might, in the displaced position of the gall-bladder, be a part of the intestines or contain large vessels, and at the depth at which they were situated it would have been exceedingly difficult, if not impossible, to control the hæmorrhage or repair the damage to the intestinal wall, should it have have proved to be intestine. Indeed, I debated seriously even abandoning the attempt to remove the stones at one time, on account of the difficulty of reaching them; and, on reviewing the case, I am inclined to believe that it would have been better to close up the abdominal wound, and trust to the possibility of the stones ulcerating into the duodenum, to which the gall-bladder was already closely matted.

The fatal issue in the man's debilitated condition was due to the shock of a prolonged operation and the moderate after-hæmorrhage.

One other point demands notice. It is very evident that the hypodermic needle-point did not touch the stones, and it is somewhat doubtful to me whether the probe passed through; the aspirator-needle did, judging from the depth at which they were found. But the sensation of scratching such a stone was so deceptive that all three of the resident-physicians, as well as myself were under the impression that the probe certainly did so, and we were fairly certain that the hypodermic needle also did.

DISCUSSION.

Dr. William Hunt: What has been the success following this operation?

Dr. Keen: I have not had time to gather the cases together; but when I prepared my paper last year there were thirty-eight cases, with eight deaths.

Dr. S. W. Gross: The percentage of deaths after cholecystotomy is eighteen. The percentage of deaths is far greater than after removal of the gall-bladder, or cholecystectotomy.

Dr. J. Ewing Mears: This case serves to confirm what every one who has had anything to do with abdominal surgery believes: that it is frequently impossible to tell what will be found in the abdomen before it is opened. This case was carefully examined before operation, and what was supposed to be the gall-bladder was outlined; but, when the abdomen was opened, this proved to be the left lobe of the liver. This may be regarded as one of the most obscure cases of cholecystotomy that has yet been performed. In the reported cases there has been no difficulty in locating the position of the gall-bladder.

Dr. W. W. Keen: In the three prior operations which I had done, there was no difficulty in finding the gall-bladder.

DEFORMITY OF THE UPPER EXTREMITIES.

Dr. W. W. Keen: I have here a patient with a deformity of the upper extremities, which I suppose is due to rickets. Precisely similar conditions I have never before seen. This girl is in her fifteenth year, a mulatto, tall and rather slender for her years. One year ago she began menstruating, and at the same time noticed the commencement of the changes which are now most distinct in the right wrist. There is also marked angular deformity at the elbow. Feeling the olecranon process, there is found considerable relaxation of the ligaments, allowing the ulna to be moved in different directions. In the wrist there is apparently no mechanism which would account for the deformity present. The deformity of the left wrist can, with the exercise of considerable force, be reduced. I would call attention to the ease with which the deformity of the elbow would be overlooked. When the hands are pronated it is not seen; but it is only observed in supination. The other bones of the body are free from evidences of rachitis. There is no trouble about the head, no beading of the ribs or enlargement at the ankles. There is no evidence of inherited specific disease: the father died of consumption, the mother is living and perfectly healthy. There is no evidence of specific disease in the condition of the teeth.

Dr. Charles W. Dulles: Would not this be more properly called a case of osteo-malacia? Rickets is an affection of the bones in the progress of their growth. Osteo-malacia is the term applied to the condition in which bones, having attained their proper development, undergo softening.

Dr. Keen: The bones are not curved to any extent, and in no other bone is there a tendency to softening.

Dr. R. J. Levis: My opinion is that the case is really a neurosis. I look on it as a paralytic condition rather than a true bone-affection. It is analogous to some forms of club-foot, and particularly to knock-knee and the like, which, we know, occur from irregular muscular action.

Dr. W. W. Keen: I cannot understand how this condition could be due to muscular action, either paralysis or contracture. There is no evidence of such a condition. No muscle is tight, and no muscle springs into relief at any point. The muscles seem to act properly, and the trouble appears to be in the bony system. In consequence of the change in obliquity, the muscles which go across as chords of the arc tend to increase the deformity.

My attention has been drawn to the diminished size of the head of the radius. This might have something to do with the deformity at the elbow, but it could have nothing to do with the condition of the wrist.

Dr. William Pancoast: I noticed that, on pressing the radius firmly against the ulna, a grating could be produced. This was only elicited on firm pressure.

